# Application of a cell microarray chip system for accurate, highly sensitive, and rapid diagnosis for malaria in Uganda

**DOI:** 10.1038/srep30136

**Published:** 2016-07-22

**Authors:** Shouki Yatsushiro, Takeki Yamamoto, Shohei Yamamura, Kaori Abe, Eriko Obana, Takahiro Nogami, Takuya Hayashi, Takashi Sesei, Hiroaki Oka, Joseph Okello-Onen, Emmanuel I. Odongo-Aginya, Mary Auma Alai, Alex Olia, Dennis Anywar, Miki Sakurai, Nirianne MQ Palacpac, Toshihiro Mita, Toshihiro Horii, Yoshinobu Baba, Masatoshi Kataoka

**Affiliations:** 1 Health Research Institute, National Institute of Advanced Industrial Science and Technology (AIST), Hayashi-cho 2217-14, Takamatsu 761-0395, Japan; 2 Panasonic Co., Automotive & Industrial Systems Company, 1006 Ooaza-Kadoma, Kadoma, Osaka 571-8506, Japan; 3 Faculty of Science, Gulu University, P.O. Box 166 Gulu, Uganda; 4 Faculty of Medicine, Gulu University, P.O. Box 166 Gulu, Uganda; 5 St. Mary’s Hospital Lacor, P.O. Box 180 Gulu, Uganda; 6 Department of International Affairs and Tropical Medicine, Tokyo Women’s Medical University, School of Medicine, 8-1 Kawada-cho, Shinjuku-ku, Tokyo 162-8666, Japan; 7 Department of Molecular Protozoology, Research Institute for Microbial Diseases, Osaka University, Osaka 565-0871, Japan; 8 Department of Molecular and Cellular Parasitology, Juntendo University School of Medicine, 2-1-1 Hongo, Bunkyo-ku, Tokyo 113-8421, Japan; 9 Department of Applied Chemistry, Graduate School of Engineering and Nagoya University, Furo-cho, Nagoya 464-8603, Japan; 10 MEXT Innovative Research Center for Preventive Medical Engineering, Nagoya University, Furo-cho, Nagoya 464-8603, Japan

## Abstract

Accurate, sensitive, rapid, and easy operative diagnosis is necessary to prevent the spread of malaria. A cell microarray chip system including a push column for the recovery of erythrocytes and a fluorescence detector was employed for malaria diagnosis in Uganda. The chip with 20,944 microchambers (105 μm width and 50 μm depth) was made of polystyrene. For the analysis, 6 μl of whole blood was employed, and leukocytes were practically removed by filtration through SiO_2_-nano-fibers in a column. Regular formation of an erythrocyte monolayer in each microchamber was observed following dispersion of an erythrocyte suspension in a nuclear staining dye, SYTO 21, onto the chip surface and washing. About 500,000 erythrocytes were analyzed in a total of 4675 microchambers, and malaria parasite-infected erythrocytes could be detected in 5 min by using the fluorescence detector. The percentage of infected erythrocytes in each of 41 patients was determined. Accurate and quantitative detection of the parasites could be performed. A good correlation between examinations via optical microscopy and by our chip system was demonstrated over the parasitemia range of 0.0039–2.3438% by linear regression analysis (R^2^ = 0.9945). Thus, we showed the potential of this chip system for the diagnosis of malaria.

Malaria, a mosquito-borne infectious disease, is one of the major human infectious diseases, there having been approximately 214 million clinical cases and 438,000 fatalities in 2015 alone^1^. For a global strategy for the control of malaria, prompt and accurate diagnosis is one of the key components^2^. Conventional light microscopy is recognized as the “gold standard” for malaria diagnosis, and it is widely used for the detection and quantification of malaria parasites. The procedure for light microscopic examination consists of the following steps: collection of a finger-prick blood sample, preparation of thin and thick blood smears, staining of the smears with Giemsa stain, and examination of them under a microscope for the detection of malaria parasites contained in the erythrocytes^3^. However, this microscopic examination of blood films with Giemsa staining is exacting and depends on a good staining technique and well-supervised technicians. Most routine diagnostic laboratories generally achieve a low detection sensitivity (average, 0.01% parasitemia) on examination according to the results from British laboratories submitted to the Malaria Reference Laboratory^4^. Even under favorable conditions for the detection of malaria parasites with excellent erythrocyte preparation and skilled technicians, the detection limit is low (0.001% parasitemia); and approximately 1 hr is required for the detection of a sufficient number of infected erythrocytes^5^,^6^. So, this conventional method is not suitable for rapid diagnosis; and it is quite difficult to detect a malaria infection by its use before the appearance of severe symptoms. Although the rapid diagnosis test (RDT) based on an immunochromatographic capture procedure using antibody was recently developed for malaria detection with easy operation and rapid detection time (20 min), the detection limit is similar to that of microscopy observation with Giemsa staining^5^,^7^,^8^. Furthermore, the possibility of false-positive and/or -negative results is well known as disadvantages of RDT. Recently, some new methods for malaria diagnosis based on flow cytometry, real-time PCR, and/or micromagnetic resonance relaxometry have been developed as laboratory methods^9^,^10^,^11^,^12^,^13^. However, some disadvantages remain, i.e., a relatively low detection limit with flow cytometry and the requirement of several hours for the detection of malaria parasites by real-time PCR. For prevention of the spread of malaria around the world, it is necessary to develop a sensitive, accurate, and convenient diagnostic system for early detection of this disease^2^.

Microchip technologies have been expected to allow high-throughput and highly sensitive analysis of the functions of individual cells^14^. In a previous study, we developed a novel high-throughput screening and analysis system using a cell microarray chip made from polystyrene with 20,944 individually addressable microchambers for the detection of malaria-infected erythrocytes from malaria cultures, one allowing ultra-high sensitivity and results within a short time^15^. This cell microarray chip was developed to allow the regular dispersion of an erythrocyte suspension in a nucleus-staining fluorescence dye in the microchambers, with the formation of a monolayer, and analysis with a commercially available DNA microarray scanner for detection of fluorescence-positive malaria nuclei in the erythrocytes. However, this system employed centrifugation to isolate erythrocytes from malaria cultures and an expensive DNA microarray scanner for the detection of fluorescence-positive malaria parasite-infected erythrocytes. For the application of a cell microarray chip system for clinical use in the field, in the present study we employed a push column with silicon oxide (SiO_2_) nano-fibers to isolate erythrocytes from whole blood and a fluorescence detector with a CCD camera instead of centrifugation and DNA microarray scanner, respectively (Fig. 1). In the present study, we showed the potential of this new cell microarray chip system for the detection of malarial parasites in blood samples taken from patients in Uganda.

## Results

### Removal of leukocytes and recovery of erythrocytes

Over 99.9% of leukocytes in a diluted blood sample could be removed by the filtration through the push column, and about 40% of the erythrocytes in the diluted blood were recovered in the inner tube. Furthermore, similar efficiency of removal of leukocytes and the recovery of the erythrocytes from blood samples from sickle cell disease patients (nos 11–15) by filtration through this push column was confirmed (Table 1).

### Dispersion of erythrocytes on the cell microarray chip

**Figure 2:**
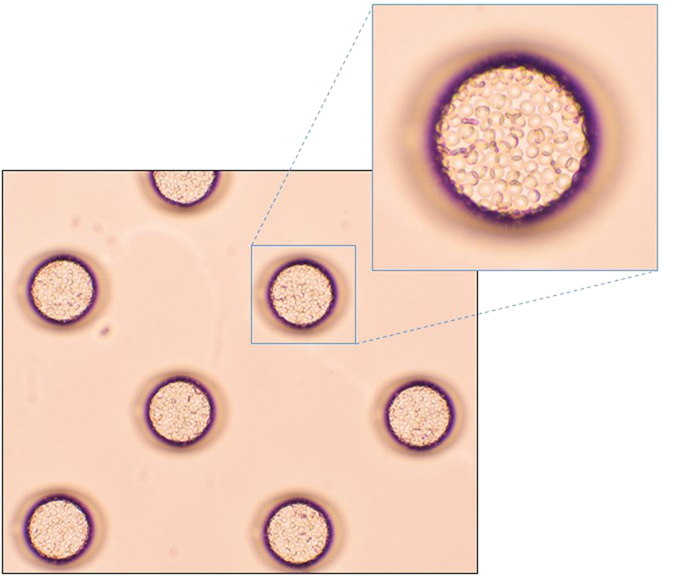
Dispersion and confinement of erythrocytes in the microchambers. Photographic light microscopic images of erythrocytes isolated from a malaria patient’s whole blood and introduced onto a cell microchip. Monolayer formation of erythrocytes in the microchamber is evident.

To achieve the confinement of erythrocytes in each microchamber, we optimized the hydrophilicity of the cell microarray chip surface by subjecting it to reactive ion-etching^15^. After passage through the push column, the erythrocytes suspended in the fluorescent nuclear dye were dropped onto a cell microarray chip by using a pipette and allowed to settle down under gravitational force and adhere to the chip surface. After washing of the chip surface with RPMI 1640 medium delivered by a pipette, only those cells that had adhered to the bottom surface of each microchamber remained as a monolayer (Fig. 2). The number of confined erythrocytes from each of 41 patients was determined by light microscopic examination of 5 different microchambers. For each patient, 122 ± 2 (mean ± standard error) to 90 ± 3 erythrocytes were quantitatively accommodated in each microchamber (data not shown).

### Determination of the percentages of parasitemia

**Figure 3:**
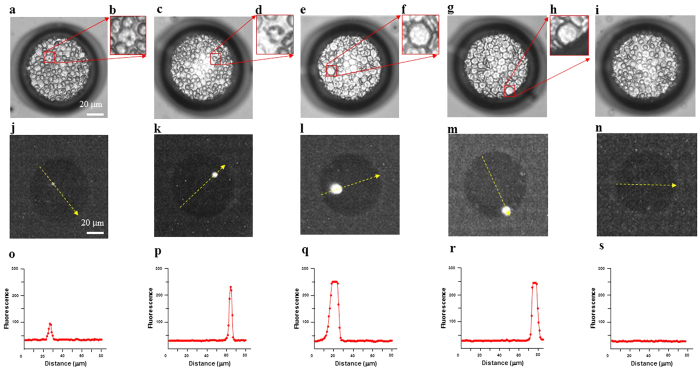
Discrimination of leukocytes and malaria parasite-infected erythrocytes in the microchamber. (**a,c**) Light microscopic images of malaria parasite-infected erythrocytes dispersed in a microchamber. Malaria parasite-infected erythrocytes were observed in the boxed regions. (**b,d**) Magnified views of the boxed regions. (**e,g**) Light microscopic images of cells in whole blood in a microchamber. A leukocyte is seen in each boxed region. (**f,h**) Magnified views of the boxed regions. (**i**) Light microscopic image of non-infected erythrocytes dispersed in the microchamber. (**j,k**) Fluorescent images of malaria parasite-infected erythrocytes in the microchamber visualized with a CCD camera. (**l,m**) Fluorescent images of leukocytes. (**n**) Image of non-infected, fluorescence-negative erythrocytes. (**o–s**) Fluorescence-intensity profile along the yellow-dotted arrow in each image shown in (**j**–**n**) respectively.

**Figure 4:**
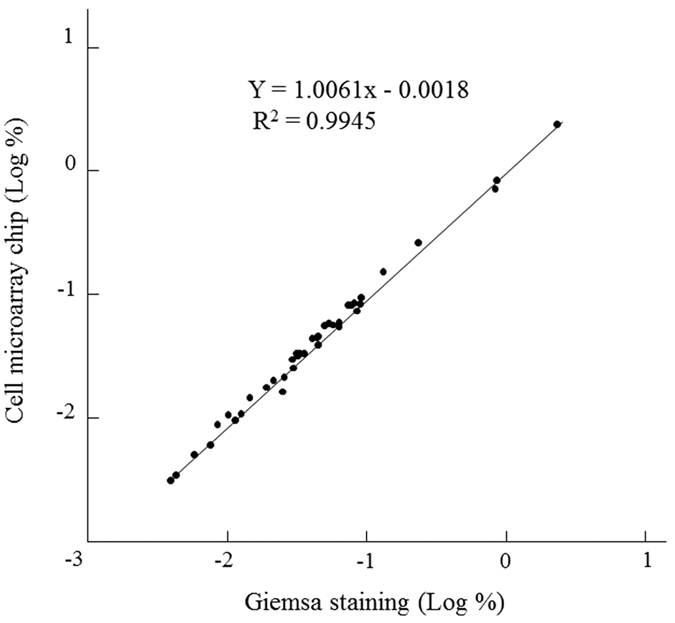
Comparative analysis of estimated parasitemia obtained by conventional microscopy examination of Giemsa-stained erythrocytes and the cell microarray chip system. Linear regression analysis was performed (R^2^ = 0.9945).

Blood samples from 41 patients were examined under a light microscope with Giemsa staining, and malaria parasites were found in 37 of them, with parasitemia ranging from 0.0039% to 2.3438% (Table 2). Malaria parasites were not found by microscopy with Giemsa staining in the remaining 4 blood samples (sample nos 21, 36, 38, 50), and so we reported them to have 0% parasitemia. We examined at least 1.5 million erythrocytes in each sample by light microscopy. By use of the cell microarray chip system, malaria parasite-infected erythrocytes were detected as being fluorescence positive, as shown in Fig. 3j,k. In the same blood samples, malaria parasite-positive erythrocytes were found, with parasitemia ranging from 0.0033% to 2.3943% (Table 2). In the same 4 samples found to show 0% parasitemia by light microscopic examination, no malaria parasite-infected erythrocytes were detected with the chip microarray system, either. As shown in Fig. 4, linear regression analysis of estimated parasitemia obtained by both methods revealed a significant relationship (R^2^ = 0.9945). By the RDT, all 41 samples were decided as positive for malaria infection. On the other hand, 40 samples were positive, and 1 sample was negative (sample no. 32), by the PCR analysis.

Fluorescence-positive erythrocytes, which were equivalent to malaria parasites, could not be detected in any of 50 healthy donors by use of the cell microarray chip system (data not shown).

### Discrimination of leukocytes and malaria-infected erythrocytes on the cell microarray chip

Bright-field images of malaria parasite-infected erythrocytes (Fig. 3a–d), leukocytes (Fig. 3e–h), and non-infected erythrocytes (Fig. 3i) corresponding to the fluorescent images were obtained. Fluorescence-positive leukocytes stained with SYTO21 in a microchamber are shown in Fig. 3(l,m), and their fluorescence intensity was apparently higher than that of the malaria parasites (Fig. 3j,k). Leukocytes could thus be easily distinguished from malaria parasite-infected erythrocytes on the basis of the large difference in fluorescence intensity and size by use of the cell microarray chip system (Fig. 3o–r), and leukocytes were automatically ignored (not counted) by the image-processing software used.

## Discussion

Earlier we reported a cell microarray chip system for highly sensitive, accurate, and rapid detection of malarial parasites in malaria cultures^15^. However, in that study we employed centrifugation and a commercially available leukocyte isolation filter (LeukoLOCK^TM^, Ambion, Inc., TX) to obtain purified erythrocytes from whole blood. Furthermore, we employed a DNA microarray scanner with confocal laser scanning for the detection of fluorescence-positive, malaria-infected erythrocytes. However, this methodology is not suitable in the field setting because of high cost, complicated technical handling, and its time-consuming aspect. In our present study, we employed a push column without any power supply and a leukocyte isolation filter instead of centrifugation, as well as a fluorescence detector with a CCD camera instead of an expensive DNA microarray scanner. Although almost all of the leukocytes (over 99.9%) in the whole blood remained in the outer tube by use of the push column, a nominal number of leukocytes were found among the isolated erythrocyte fraction on the cell microarray chip. The condensability of leukocyte nuclei is apparently greater than that of nuclei of malaria parasites, as reported previously^15^. In the present study, the fluorescence intensity of the leukocytes was higher than that of the parasites, indicating greater nuclear condensability, as was shown in Fig. 3. Thus, it was easy to distinguish between malaria parasite-infected erythrocytes and leukocytes by comparing their fluorescence intensities by using this newly developed cell microarray chip system.

It required over 22 min to scan a whole cell microarray chip (20,944 microchambers, 112 clusters) for the detection of malaria parasite-infected erythrocytes by using the fluorescent detector with CCD camera (data not shown). A reduction in diagnosis time is important in field use, and so we only scanned 25 clusters (4675 microchambers), requiring only 5 min. In our previous study, each microchamber accommodated about 130 erythrocytes from a healthy donor, and so 600,000 erythrocytes in total would be expected to be analyzed on a cell microarray chip when only 25 clusters are examined^15^. Most routine diagnostic laboratories generally achieve a low sensitivity of detection (average, 0.01% parasitemia)^4^. So we achieved a sufficiently higher level of sensitivity for malaria detection than the one found with the gold standard even when only 25 clusters examined. Although quantitative confinement of erythrocytes in each patient was observed, the number of confined erythrocytes in a microchamber differed among the respective patients. The reason for this phenomenon is not clear. In our previous study, partial loss of erythrocytes confined in the microchamber after washing of the cell microarray chip surface was observed when albumin was added to the erythrocyte fraction. We obtained blood samples from 41 patients who were suspected of having malaria at the outpatient care section of the hospital. Apparent hyperproteinemia through dehydration by high fever, which is one of the typical symptoms of malaria, may have been the reason for the loss of erythrocytes in the microchamber. Presently, 40.8 × 10^4^ to 57.6 × 10^4^ cells were examined in 25 clusters on the cell microarray chip. These numbers of erythrocytes were still sufficient to obtain high-sensitive detection of malaria parasites.

In the present study, the detection limit in the cell microarray chip system was 0.0033%, and this sensitivity was greater than that of most routine diagnostic laboratories using Giemsa staining (0.01%). Although there were no malaria parasite-infected erythrocytes in 4 samples (nos 21, 36, 38, and 50) by Giemsa staining or by use of the cell microarray chip, we did obtain positive results for these 4 samples by the RDT and/or PCR method. False positivity is known as one of the disadvantages of RDTs^5^, and the presence of residual antigenemia resulting in persistent positive results obtained by HRP2-based RDTs after a successful malaria treatment has been reported^16^. These 4 patients were under medical treatment with artemisinin. Furthermore, a positive result obtained by PCR analysis after clearance of malaria has also been reported^16^. False- positives may be due to the presence of residual malaria DNA fragments in the blood. The cell microarray chip system revealed that there were no malaria parasite-infected erythrocytes in these 4 samples. Furthermore, we could not find any fluorescence-positive cells in blood samples from 50 healthy donors when the cell microarray chip was used. So there were no false-positives for malaria detection with this microarray chip system. In the PCR analysis, malarial parasite DNA was not found in sample no. 32. Malarial DNA for PCR analysis was extracted from blood blotted on a filter paper, and the reason of the failure of malaria detection may have been insufficient recovery of DNA from the filter paper. No false-negatives were found with the cell microarray chip system. From these results, accurate and quantitative malaria detection in a short time can be expected by using the cell microarray chip system with a push column to isolate erythrocytes from whole blood. For the artemisinin-based combination therapy (ACT), parasite-based *P. falciparum* diagnosis is recommended for malaria parasite-infected patients^16^. Employment of appropriate malaria diagnosis is important to prevent the overuse of ACT, reduce cost, minimize the development and spread of anti-malarial drug resistance, and to improve the management of other causes of fever^16^,^17^. So, this cell microarray chip system would also be expected to improve the outcome by virtue of the proper use of ACT. Although this system is easy to operate, and the inclusive costs of a cell microarray chip, fluorescent dye, and a push column are less than US$ 2.0, the cost of a fluorescence detector is drastically higher, being about US$ 8,000. Furthermore, the fluorescence detector is huge and requires an electric supply, as would be available in the hospital laboratory, thus making it unsuitable for field use. However a fluorescence detector is now being improved to have a more compact size, to be battery driven, and to have a lower cost for field use. Malaria parasite-infected erythrocytes could be detected at the single cell level in the cell microarray chip, and at least 10 times higher sensitivity than that achieved with Giemsa is expected (1 malaria-infected erythrocyte in 600,000 erythrocytes). Furthermore, daily parasite counting until clearance of the trophozoites has been achieved is recommended for assessing the response of malaria to treatment^17^. The present cell microarray chip system is also suitable for malaria therapeutic monitoring at the single-cell level and for quantitative detection of malaria. Therefore, we have demonstrated the potential of our cell microarray chip system using a push column for clinical diagnosis and drug monitoring with high accuracy for quantitative detection of malaria parasite-infected erythrocytes.

## Methods

### Blood samples from malaria patients and conventional diagnosis of malaria

Peripheral venous blood was obtained from 41 patients who were suspected of having malaria or who were receiving malarial treatment at the outpatient care of the Lacor Hospital at Gulu, Uganda. Blood samples positive for *Plasmodium falciparum* infection (n = 41), as determined by use of SD BIOLINE Malaria Ag *P.f* (STANDARD DIAGNOSTICS, INC., Gyeonggi-do, Republic of Korea) as a rapid diagnosis test (RDT), were examined for the presence or absence of *P. falciparum*-infected erythrocytes with conventional Giemsa-staining microscopy and with PCR. This RDT is a lateral flow chromatographic immunoassay for the quantitative detection of *P. falciparum*-specific protein, i.e., histidine-rich protein II (HRP2).

Four microliters of whole blood from malaria patients was smeared so as to produce a thin film on a slide. Each slide was stained with 5% Giemsa (Merck, Co., Ltd., Germany) stain in phosphate-buffered saline (pH7.2), and then examined under a light microscope (Olympus, Co., Ltd., Tokyo, Japan) at a magnification of x 1,000 for the counting of malaria parasites^3^. Thin smears were examined thoroughly by 2 independent, experienced microscopists who were unaware of the cell microchip results. For the examination of the presence of sickle cells, microscopic examination of erythrocytes in 1% Na_2_S_2_O_5_ solution was performed^18^.

Malaria DNA was extracted from blood blotted onto a filter paper (Whatman 3 MM Chr Blotting papers, Florham Park, NJ, USA) by using a DNA extraction kit (QIAGEN QIAmp DNA Mini Kit, Germany) according to the manufacturer’s instructions. Nested PCR was performed to confirm the presence of malaria in the blood samples. Sequences of the oligonucleotides used as primers for nested PCR were based on the *Plasmodium* small subunit ribosomal RNA genes^19^. The sequences and the position of the genus-specific primers rPLU 6 and rPLU 5 were the following: rPLU 6,5′-TTAAAATTGTTGCAGTTAAAACG-3′ (606–628); rPLU 5,5′-CCTGTTGTTGCCTTAAACTTC-3′ (1736–1716); and these were used in the 1st PCR. For the specific amplification of *P. falciparum*, rFAL 1,5′-TTAAACTGGTTTGGGAAAACCAAATATATT-3′ (664–693) and rFAL 2,5′-ACACAATGAACTCAATCATGACTACCCGTC-3′ (869–840) were employed as primers for the 2nd PCR. For both PCRs, 1.0 μl of DNA sample was mixed with Ex *Taq* buffer (Takara Bio Inc., Siga, Japan) containing 0.2 μl of each primer, 2 mM MgCl_2_, a 0.2 mM concentration of each deoxynucleoside triphosphate, and 0.5 U Ex *Taq* (Takara Bio Inc.) in a final volume of 20 μl. Conditions for the first PCR were as follow: step 1, 95 °C for 4 min; step 2, denaturation at 94 °C for 1 min; step 3, annealing at 58 °C for 2 min; step 4, extension at 72 °C for 2 min; step 5, 25 repeats of steps 2–4, and 72 °C for 3 min. Those for the second PCR were as follow: step 1, 95 °C for 5 min; steps 2, denaturation at 94 °C for 1 min; step 3, annealing at 58 °C for 2 min; step 4, extension at 72 °C for 2 min; step 5, 30 repeats of step 2–4, and 72 °C for 5 min.

### Isolation of erythrocytes from whole blood by using the push column for cell microchip analysis

**Figure 5:**
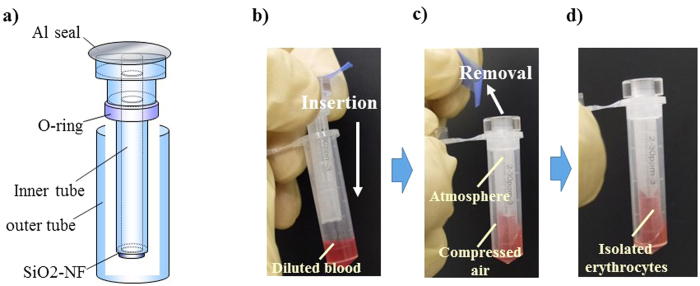
Push column for erythrocyte isolation from whole blood. (**a**) A push column was employed for the isolation of erythrocytes from whole blood. (**b–d**) A mixture of 6 μl of whole blood and 294 μl of RPMI 1640 was poured into the outer tube, and the inner tube was then pushed into the outer tube. Erythrocytes were recovered in the inner tube by filtration through SiO_2_-nano-fibers, and leukocytes remained in the outer tube. The usage of these images was licensed by Panasonic Co., AIS Co.

The push column (EZBNPP01AT, Panasonic Co., Osaka, Japan) was employed for the isolation of erythrocytes from whole blood (Fig. 5). Briefly, a mixture of 6 μl of whole blood and 294 μl of RPMI 1640 (Nacalai Tesque, Inc., Tokyo, Japan) was poured into the outer tube; and then the inner tube was pushed into the outer tube. Erythrocytes were recovered in the inner tube by filtration through SiO_2_-nano-fibers, and the leukocytes remained in the outer tube. To confirm the efficiency of erythrocyte recovery and removal of leukocytes from whole blood, we counted the numbers of erythrocytes and leukocytes with a hemocytometer before and after the filtration of whole blood with this push column. The efficiency of erythrocyte isolation and removal of leukocytes was calculated as follows: number of erythrocytes in the eluted portion/number of erythrocytes in whole blood × 100(%) and number of leukocytes in whole blood − number of leukocytes in the eluted portion/ number of leukocytes of whole blood × 100(%), respectively.

### Construction of the cell microarray chip

**Figure 6:**
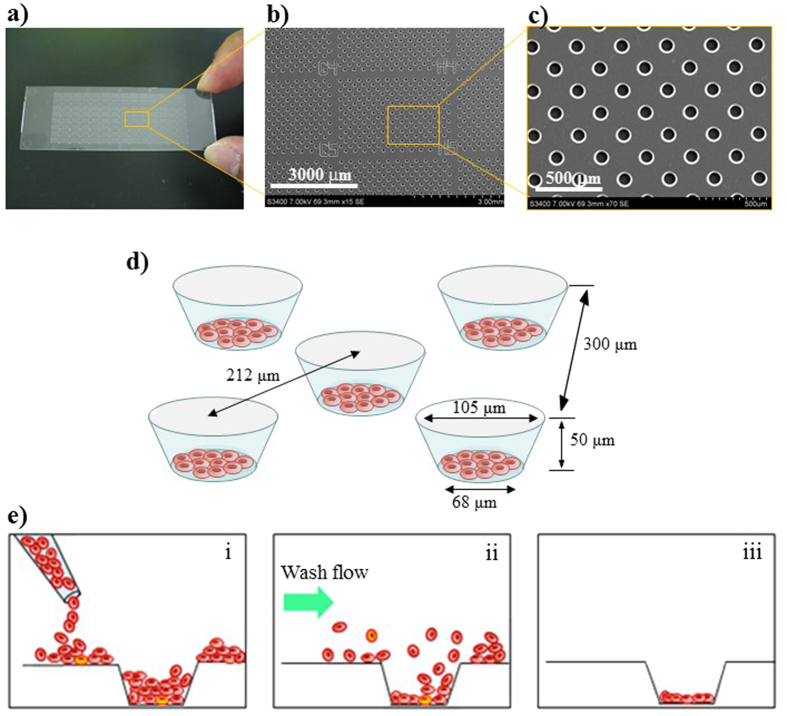
Construction of the cell microarray chip and monolayer formation of erythrocytes in the microchamber. (**a**) Photographic and (**b,c**) SEM images of a cell microarray chip. The cell microarray chip comprised 20,944 microchambers in a plastic slide of glass-slide size. The cell microarray chip had 112 (14 × 8) clusters of 187 microchambers. (**d**) Each microchamber was 105 μm in width, 50 μm in depth, and 300 μm in pitch, and comprised a frustum with a 68-μm diameter flat bottom for the accommodation of erythrocytes as a monolayer. These images were cited from doi: 10.1371/journal.pone.0013179.g002. (**e**) Schematic cross-section images of erythrocytes in microchamber, showing (i) dispersing, (ii) washing for removing the excess erythrocytes, and (iii) monolayer formation.

As previously reported, a cell microarray chip with 20,944 microchambers (105-μm width, 50-μm depth, and 300-μm pitch) was made from polystyrene by the Lithographie Galvanoformung Abformung process by Starlight Co. Ltd. (Osaka, Japan, Fig. 6a–c)^15^. The cell microarray chip was fabricated by injection molding with a nickel mold. The cell microarray chip had 112 (14 × 8) clusters, each with 187 microchambers. Block numbers were put on the clusters for easy confirmation of malaria-infected erythrocytes by light microscopic observation after fluorescence scanning. Each microchamber had the shape of a frustum (Fig. 6d). To achieve erythrocyte confinement in the microchambers, we rendered the cell microarray chip surface hydrophilic by means of reactive ion-etching treatment using the SAMCO RIE system (SAMCO, Inc., Tokyo, Japan). The effect of reactive ion-etching exposure on the cell microarray chip surface was examined by measuring the contact angle of water on the chip surface with a contact-angle meter (Kyowa Interface Science Co., Ltd., Saitama, Japan)^20^.

### Detection of malaria parasites with the cell microarray chip system

For the detection of malaria parasites on a cell microarray chip, 3.1 μl of 50 μM SYTO 21 (Life Technology, Co., CA), which is a nucleus-specific fluorescence dye (Ex: 494 nm, Em: 517 nm) was added to 250 μl of isolated erythrocytes, and the mixture was dispersed manually onto the cell microarray chip by using a pipette, followed by 10 min of standing, to allow the erythrocytes to settle down into the microchambers under gravitational force. Then the excess erythrocytes on the cell microarray chip surface were removed by gentle washing with RPMI 1640 medium (Fig. 6e). Twenty-five clusters on the cell microarray chip were scanned with a fluorescence detector attached to a CCD camera system (EZBLMLH01T, Panasonic Co.). For 5-min scanning, a 488-nm semiconductor laser, objective lens, optimal filter, and XYZ axis electric stage were used. For all cell microarray chip analysis, 25 randomly selected clusters were examined. For detection of the malaria parasite-infected erythrocytes, the cell microarray chip was set against the fluorescence detector; and the “analyze” button was pushed to detect the fluorescence-positive erythrocytes. This system exhibited a resolution of 1.1 μm. Malaria parasite-infected erythrocytes were distinguished from uninfected ones by the fluorescence intensity and the shape of the fluorescent spot of the former. These fluorescence parameters of individual erythrocytes were determined with attached image processing software; and the erythrocytes that exhibited fluorescence intensities of 1.3 times above and 7.5 times below that of uninfected erythrocytes were considered to be malaria parasite-infected erythrocytes. When the aspect ratio of the fluorescent spot was above 2.8 or its area was below 4.8 μm^2^ or above 145 μm^2^, this case was taken to be the detection noise.

The percentage of parasitemia in each patient was determined as follows: [the number of erythrocytes detected as malaria parasite-infected ones by the fluorescence detector/the average number of erythrocytes confined in microchamber × 187 (microchambers) × 25 (clusters)] × 100.

We also obtained blood samples from 50 Japanese volunteers who had never been to a malaria-endemic area and were negative by RDT. These blood samples were also analyzed by using the cell microarray chip to examine the presence or absence of fluorescence-positive erythrocytes according to the above-mentioned procedure.

### Ethics

This study was approved by the Institutional Review Board of the National Institute of Advanced Industrial Science and Technology for the use of human derivatives for biomedical research, and by the Ethics Committee of Uganda National Council for Science and Technology. All subjects provided written informed consent for the collection of samples and subsequent analysis. All experiments were carried out in accordance with the approved guidelines.

## Additional Information

**How to cite this article**: Yatsushiro, S. *et al.* Application of a cell microarray chip system for accurate, highly sensitive, and rapid diagnosis for malaria in Uganda. *Sci. Rep.* **6**, 30136; doi: 10.1038/srep30136 (2016).

## Figures and Tables

**Figure 1:**
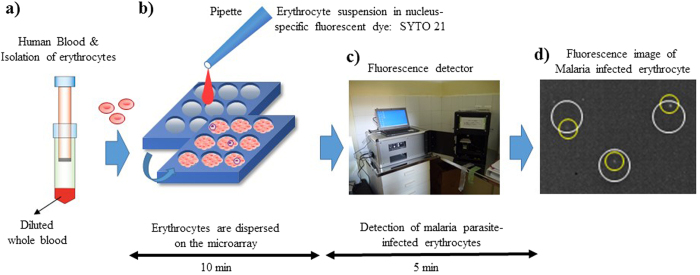
Schematic process for detection of malaria-infected erythrocytes by using a cell microarray chip system. (**a**) Erythrocytes were isolated from whole blood by using a push column. (**b**) Erythrocytes stained with a nucleus-specific fluorescent dye, SYTO 21, for the staining of malaria nuclei were dispersed on a cell microarray chip by using a pipette, which led to the formation of a monolayer of erythrocytes in the microchambers. (**c**) Malaria parasite-infected erythrocytes were detected by using a fluorescence detector for monitoring fluorescence-positive erythrocytes. (**d**) The target malaria parasite-infected erythrocytes were analyzed quantitatively at the single-cell level (white circle: microchamber, yellow circle: malaria parasite-infected erythrocyte).

**Table 1:** Recovery of erythrocytes and removal of leukocytes from whole blood by use of the push column.

No.	Before the filtration	After the filtration	Removal rate of leukocytes (%)	Recovery rate of erythrocytes (%)
	Number of cells/μl Leukocytes/Erythrocytes	Number of cells/μl Leukocytes/Erythrocytes		
1	7500/4518000	2/1984000	99.97	43.91
2	7500/4518000	0/2058000	100	45.56
3	7500/4518000	1/1984000	99.99	43.91
4	4500/4030000	0/2070000	100	51.36
5	3750/4900000	0/2640000	100	53.88
6	1750/2060000	0/930000	100	45.15
7	3500/3030000	3/1230000	99.91	40.59
8	4250/4770000	4/2500000	99.90	52.41
9	4250/4770000	3/2500000	99.93	52.41
10	5125/3850000	5/2160000	99.98	56.10
11	7125/5170000	5/2620000	99.93	50.68
12	4750/2710000	0/1550000	100	57.20
13	4500/3060000	0/1770000	100	57.84
14	4500/3060000	3/1530000	99.93	50.00
15	7125/5170000	5/2150000	99.93	41

**Table 2:** Accuracy of the cell microarray chip system for the detection of malaria parasite-infected erythrocytes.

No.	Giemsa staining (%)	Number of parasites/erythrocytes	Cell microarray chip (%)	Number of parasites/erythrocytes	RDT	PCR
16	0.0042	42/1002216	0.0034	16/467976	+	+
17	0.0429	129/300663	0.0386	170/440440	+	+
18	0.0292	88/301914	0.0300	144/480216	+	+
19	0.0600	180/301619	0.0585	293/500559	+	+
20	0.0249	75/301699	0.0230	101/439022	+	+
21	0.0000	0/1504523	0.0000	0/499664	+	+
22	0.0402	121/300811	0.0460	248/538802	+	+
23	0.0123	123/1001167	0.0109	55/505306	+	+
24	0.0101	101/1001526	0.0105	48/456280	+	+
25	0.0904	91/100667	0.0838	394/470157	+	+
26	0.0488	149/305578	0.0576	272/472630	+	+
27	0.0039	39/1000895	0.0033	17/520750	+	+
28	0.8639	261/30211	0.8596	4596/534670	+	+
29	0.0299	90/300887	0.0259	140/540751	+	+
30	0.2467	84/31733	0.2231	972/435681	+	+
31	0.0535	161/301217	0.0597	273/457028	+	+
32	0.0359	108/301183	0.0337	165/489475	+	—
33	0.0636	192/302113	0.0557	262/470462	+	+
34	0.0423	127/300284	0.0458	234/511261	+	+
35	0.0146	132/906348	0.0168	76/452497	+	+
36	0.0000	0/1506942	0.0000	0/561456	+	+
37	0.0256	77/301138	0.0174	80/459427	+	+
38	0.0000	0/1502416	0.0000	0/513128	+	+
39	0.0319	100/313472	0.0320	141/440241	+	+
40	0.0329	99/301265	0.0342	174/508640	+	+
41	2.3438	249/10624	2.3943	11867/495643	+	+
42	0.1320	134/101521	0.1320	611/462874	+	+
43	0.8566	293/34205	0.7425	3237/435941	+	+
44	0.0081	82/1006147	0.0094	39/417059	+	+
45	0.0974	118/121149	0.1012	441/435771	+	+
46	0.0211	85/402843	0.0218	104/477066	+	+
47	0.0061	61/1000192	0.0058	25/433952	+	+
48	0.0297	111/373259	0.0301	163/541150	+	+
49	0.0044	44/1011262	0.0046	24/516795	+	+
50	0.0000	0/1501671	0.0000	0/576102	+	+
51	0.1000	120/120036	0.0925	438/473428	+	+
52	0.0909	72/79241	0.0984	401/407711	+	+
53	0.0820	98/119524	0.0880	434/493375	+	+
54	0.0101	57/567101	0.0091	46/503772	+	+
55	0.0635	165/259724	0.0602	272/451468	+	+
56	0.8611	251/29148	0.8715	3813/437511	+	+
